# Vascular stem cells in diabetic complications: evidence for a role in the pathogenesis and the therapeutic promise

**DOI:** 10.1186/1475-2840-11-37

**Published:** 2012-04-23

**Authors:** Emily C Keats, Zia A Khan

**Affiliations:** 1 Department of Pathology, University of Western Ontario, London, ON, Canada; 2 Metabolism and Diabetes Program, Lawson Health Research Institute, London, ON, Canada; 3 4011 Dental Sciences Building, 1151 Richmond Street, London, ON, N6A 5C1, Canada

**Keywords:** Diabetes, Diabetic complications, Angiopathy, Endothelial cells, Vasculogenesis, Angiogenesis, Stem cells, Progenitors, Perivascular cells

## Abstract

Long standing diabetes leads to structural and functional alterations in both the micro- and the macro-vasculature. Vascular endothelial cells (ECs) are the primary target of the hyperglycemia-induced adverse effects. Vascular stem cells that give rise to endothelial progenitor cells (EPCs) and mesenchymal progenitor cells (MPCs) represent an attractive target for cell therapy for diabetic patients. A number of studies have reported EPC dysfunction as a novel participant in the culmination of the diabetic complications. The controversy behind the identity of EPCs and the similarity between these progenitor cells to hematopoietic cells has led to conflicting results. MPCs, on the other hand, have not been examined for a potential role in the pathogenesis of the complications. These multipotent cells, however, do show a therapeutic role. In this article, we summarize the vascular changes that occur in diabetic complications highlighting some of the common features, the key findings that illustrate an important role of vascular stem cells (VSCs) in the pathogenesis of chronic diabetic complications, and provide mechanisms by which these cells can be used for therapy.

## Chronic diabetic complications

Diabetes is a chronic and debilitating metabolic disease that presently has no cure. Currently, the total number of people with diabetes is upwards of 221 million and in North America alone, more than 10% of the population is affected [1]. This amounts to a staggering economic burden, estimated to reach $17 billion a year by 2020 in Canada [2], and almost $116 billion in the United States [3]. Although the incidence in North America is quite alarming, close to 80% of diabetes-related deaths occur in low- and middle- income countries due to poor management of complications and lower standards of healthcare [4]. Despite great efforts to combat this disease, the World Health Organization projects that diabetes-related deaths will more than double by the year 2030 [4].

The most important discovery in the diabetes field was that of insulin in 1921. Exogenous insulin significantly alleviated diabetic coma and ketoacidosis, and saved millions. However, diabetic patients are still not morbidity-free due to the chronic secondary complications that arise in every diabetic patient. These long-term complications manifest as micro- (retinopathy, neuropathy, nephropathy, and cardiomyopathy) and macro- (atherosclerosis) vascular dysfunctions [5]. Although the clinical features of the complications are quite varied, the underlying cause is an aberration in the vasculature of the target organs. Two major clinical trials paved the way to better understanding the cause of the diabetic complications: the Diabetes Control and Complications Trial (DCCT) and the United Kingdom Perspective Diabetes Study (UKPDS), completed in 1993 and 1997 respectively. In both trials, type 1 and type 2 diabetic patients were put under intensive glycemic control, and in both cases, there was delayed progression and/or inhibition of the onset of diabetic complications [6,7]. It is true that other factors, such as hyperlipidemia and hyperinsulinemia, may contribute to the pathogenesis of diabetic complications. The results of the clinical trials and years of research in animal models of diabetes and cultured cells confirm the notion that hyperglycemia is the primary cause of the micro- and macro-angiopathy we see in long-term diabetes.

## Molecular basis of the vascular dysfunction in diabetic complications

**Figure 1:**
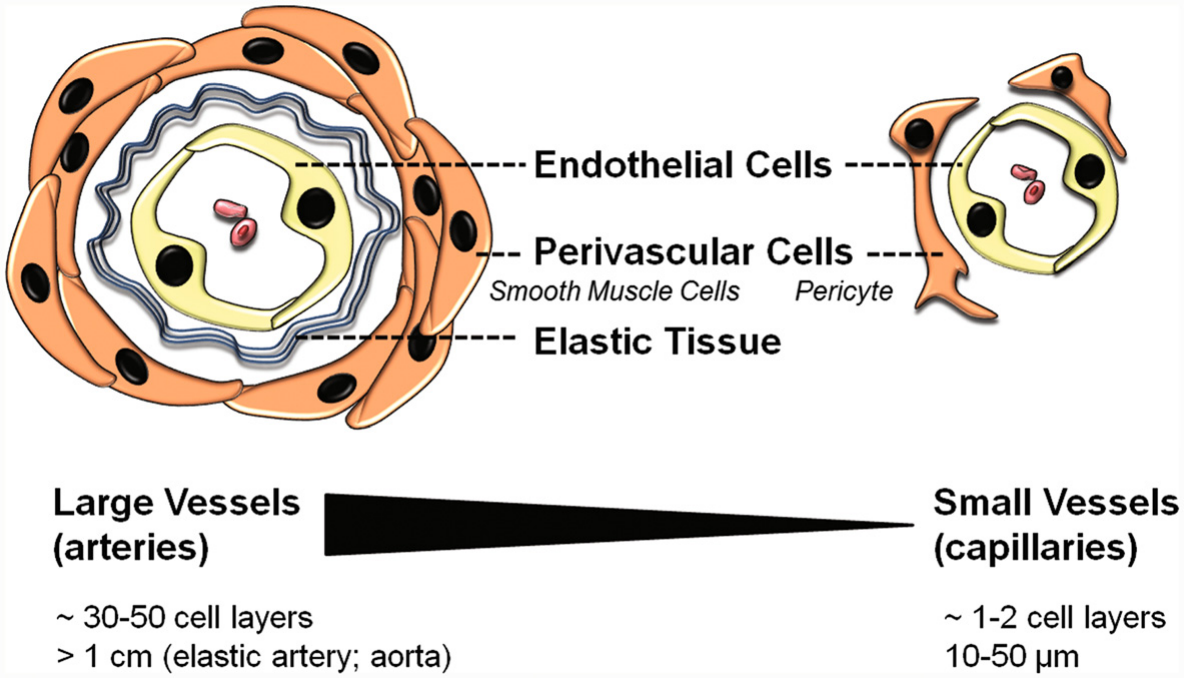
**Schematic of large and small blood vessels.** Large vessels contain a prominent elastic tissue and multiple layers of the contractile cells (smooth muscle cells). Small capillaries may or may not have contractile cell (pericyte) coverage. Endothelial cells in capillaries sit on a thin basement membrane.

Endothelial cells (ECs) are a critical component of the vascular unit. These specialized cells form the inner lining of blood vessels, sit on a basement membrane, and are surrounded by supportive perivascular cells (pericytes or smooth muscle cells) (Figure 1). ECs not only function as a barrier- producing an interface between circulating blood and the perfused tissue- but also play a prominent role in tissue functioning as well as organogenesis. These cells are involved in various important vascular processes such as regulating blood flow and pressure, permeability, blood fluidity, the thrombotic/fibrinolytic balance, and leukocyte traffic [8,9].

Due to their anatomical location in the blood vessel, ECs are not surprisingly the first to encounter circulating glucose. Glucose transporters (Gluts) facilitate the uptake of glucose [10-12]. The predominant Glut in the vascular ECs is Glut1 [13,14]. Although Gluts are typically expressed in a tissue-specific manner, Glut1 is ubiquitously expressed under normal growth conditions [15]. Unlike many other glucose transporters, Glut1 activity and expression level does not change with increased or decreased plasma glucose levels. This indicates that hyperglycemia may have profound detrimental effects on vascular ECs specifically, as glucose uptake may not be actively regulated [13,14]. There are certain conditions, however, that may alter Glut1 expression. For example, hypoxia increases Glut1 levels in ECs [16]. This may be one of the mechanisms behind uncontrollable dysfunction of the ECs in diabetic complications.

*In vitro* studies have shown that exposure to high levels of glucose lead to biochemical alterations in mature vascular ECs [17]. These alterations manifest as increased production of extracellular matrix proteins, such as collagen and fibronectin, increased production of the procoagulant protein von Willebrand Factor (vWF), and altered cellular activities [18-20]. In addition to a reduction in proliferation and migration [21], a number of studies have provided evidence that hyperglycemia can directly promote EC apoptosis [22-24]. This apoptotic pathway is believed to be activated by increased oxidative stress, increased intracellular Ca^2+^, mitochondrial dysfunction, changes in intracellular fatty acid metabolism, activation of mitogen activated protein kinase (MAPK) signaling pathways, and impaired phosphorylation/activation of protein kinase B (also known as Akt) [25,26].

**Figure 2:**
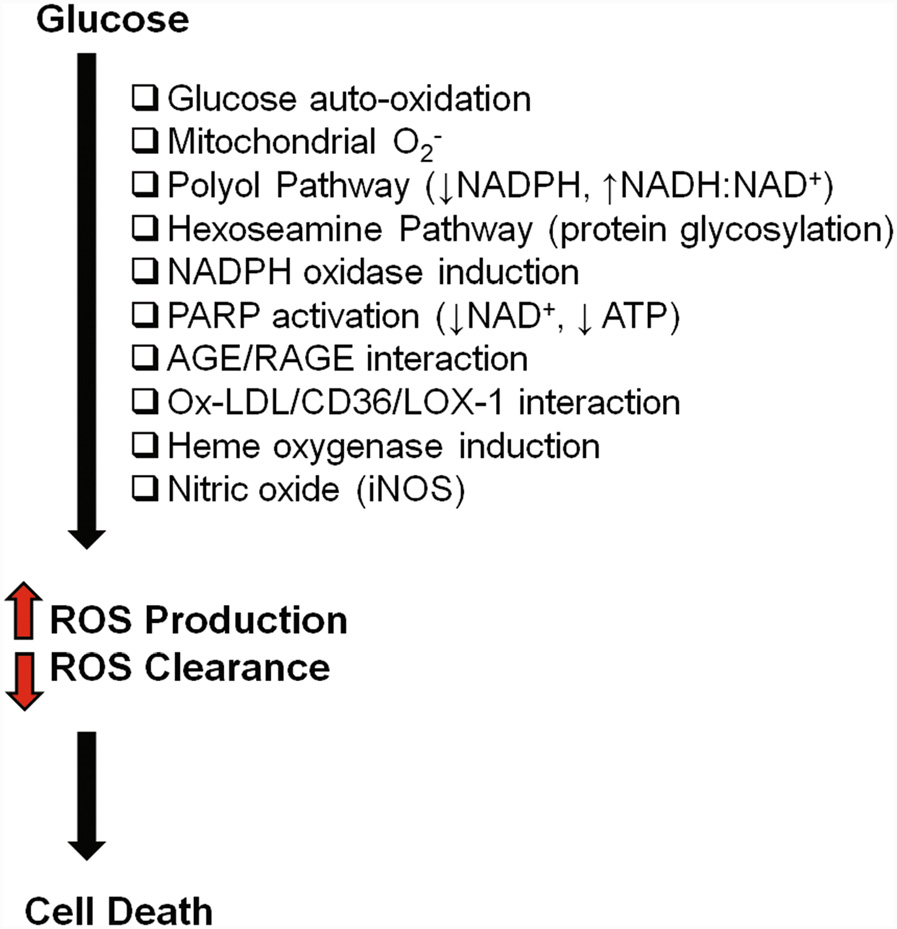
**Mechanisms of glucose-induced oxidative stress in ECs.** Hyperglycemia leads to cell death by the overproduction of ROS and impairment in the ROS neutralizing enzymes. Multiple pathways may lead to ROS production. A consequence of activating these oxidant pathways is the depletion of co-factors required by the anti-oxidant enzyme systems. The net effect is an imbalance in ROS production and ROS clearance [AGE=advanced glycation end product; ATP=adenosine-5'-triphosphate; iNOS=inducible nitric oxide synthase; LOX-1=receptor for oxidized-low density lipoprotein; NAD=nicotinamide adenine dinucleotide; NADPH=reduced NAD phosphate; ox-LDL=oxidized-low density lipoprotein; PARP=poly (ADP-ribose) polymerase; RAGE=receptor for AGE].

One of the earliest functional changes, which precedes any structural change in the vasculature of the target organs, is the impairment of endothelial-dependent vasodilation [18]. This impairment arises because of two inter-regulated mechanisms: decreased production of vasodilators and increased production of vasoconstrictors. Diminished levels of nitric oxide (NO) and increases in endothelin-1 (ET-1), the most potent endogenous vasoconstrictor, have been demonstrated in vascular ECs cultured in high glucose [18]. We and others have shown that the enzymes involved in NO production are upregulated in the ECs upon glucose challenge [27]. However, uncoupling of the enzymatic reaction and possible sequestration of NO by oxidative stress leads to significantly reduced NO [18,28]. Another well-established pathway leading to increased EC damage in diabetes is the oxidative stress pathway. Hyperglycemic ECs produce reactive oxygen species (ROS) such as hydroxyl radicals, superoxide anions, and hydrogen peroxide [19]. The overproduction of ROS may also be attributed to the activation of alternate metabolic/signaling pathways such as the polyol pathway and hexoseamine pathway, and signaling through protein kinase C, AGE formation, and PARP activation [17,29,30] (Figure 2). Each of these pathways may potentiate each other, culminating in increased ET-1 activity, reduced NO bioavailability, oxidative stress, and EC dysfunction. Remarkably, the biochemical changes that we see in high glucose-treated ECs are reminiscent of the chronic complications that present in the diabetic patients.

**Figure 3:**
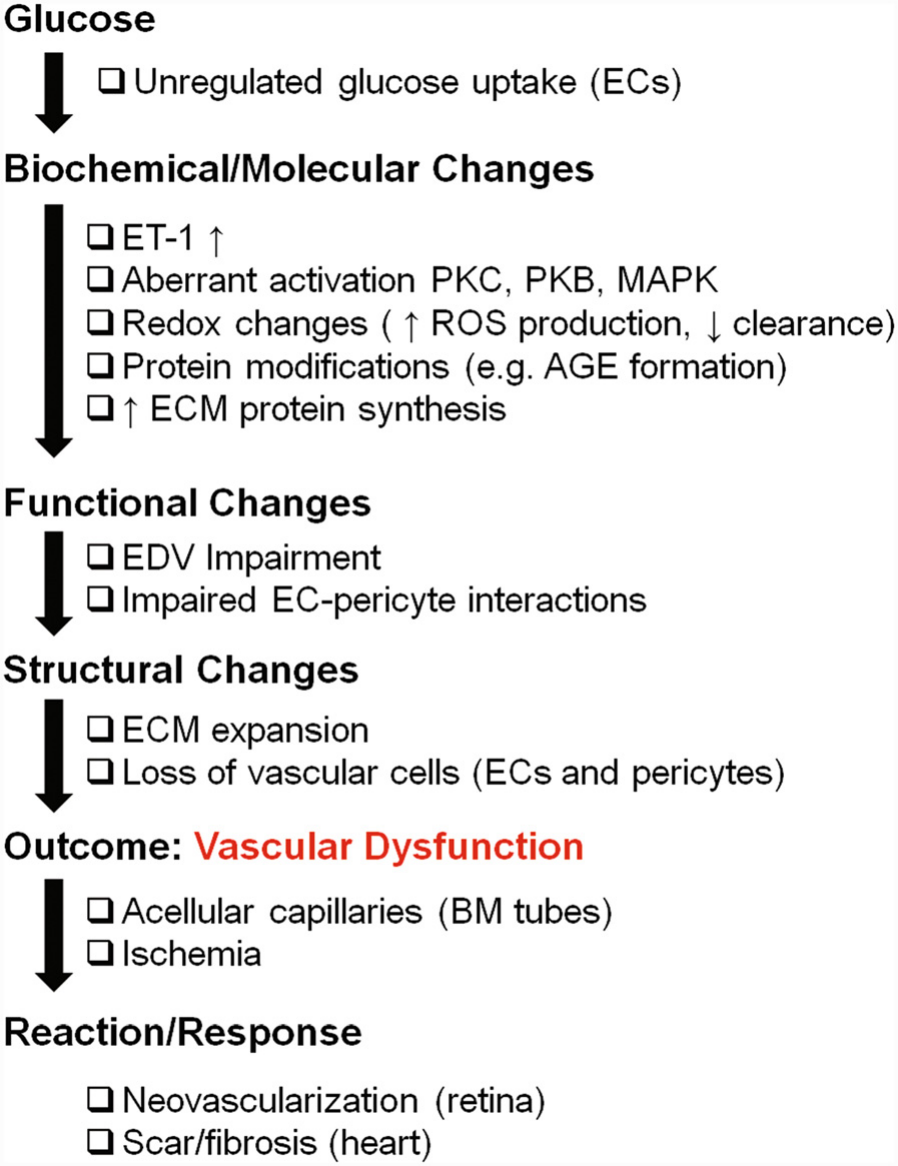
**Mechanisms leading to vascular disruption in diabetes.** High glucose causes various biochemical and molecular changes in the vascular ECs, resulting in functional and structural alterations of the target organ vascular bed. Impaired vasoregulation and loss of vessel integrity leads to reduced blood flow and ischemia. In response, the target organ exhibits neovascularization (diabetic retinopathy/nephropathy) or fibrosis (diabetic cardiomyopathy/neuropathy) [AGE=advanced glycation end product; BM=basement membrane; EC=endothelial cell; ECM=extracellular matrix; EDV=endothelial-dependent vasodilation; ET-1=endothelin-1; MAPK=mitogen-activated protein kinase; PKB=protein kinase B; PKC=protein kinase C].

We now know that altered ECs provide a backbone for the long-term vascular dysfunctions that arise in the diabetic patients (Figure 3). Changes in the structure and function of ECs leads to subsequent aberration of entire vascular networks, and tissues will begin to shows signs of poor blood flow and ischemia [31]. Normally, an adaptive response would be expected under these conditions. There is a vascular response in diabetic patients, although it varies depending on the organ system involved. For example, the retina and kidneys typically exhibit enhanced blood vessel formation, while this process is impaired in the heart and lower limbs [31,32]. The selectivity in the target organ system in diabetes suggests the importance of both the tissue microenvironment and the intrinsic properties of the ECs [18].

Growth factors and extracellular matrix (ECM) proteins are two major regulators of the balance that exists between neovascularization and scar formation/fibrosis in diabetic complications. Vascular endothelial growth factor (VEGF) is an EC-specific mitogen that promotes angiogenesis in a number of disease models. In parallel with a lack of angiogenesis that occurs in the heart in diabetes, a reduced expression of VEGF and its receptors is reported in the myocardium [33]. This is in direct contrast to elevated VEGF levels in the retina [34], correlating with uncontrolled retinal neovascularization. In addition to growth factors, the ECM regulates the vascular cells and may contribute to the differential effects of high glucose levels in diabetic complications. Binding of EC surface integrins to the ECM proteins regulates cell survival/apoptosis, growth, and cytoskeletal changes [35]. Therefore, angiogenesis is highly dependent on the interactions between the cellular components of the vascular unit and the surrounding scaffolding proteins. In fact, ECM changes that are believed to promote neovascularization during tumorigenesis are mimicked in retinal vascular development, and include increases in fibronectin and laminin [36]. Retinal basement membranes of diabetic animals show a similar protein profile- with elevated collagen IV, laminin and fibronectin as early as 8weeks following the onset of diabetes [37]. Along with ECM protein heterogeneity, increased ECM deposition in the heart may contribute to the impaired angiogenic response. Cardiac fibroblasts, which are present in significant numbers, may be responsible for this unregulated deposition of ECM proteins through the action ET-1 which has been shown *in vitro* to increase production of ECM components by fibroblasts [38,39].

Regardless of the organ system, vascular ECs are the primary mediators of hyperglycemic damage, and they undergo functional and structural changes. Subsequent impaired vasoregulation, increased permeability, ECM expansion, and dysfunction of entire vascular networks causes reduced blood flow to the target organ, setting the stage for uncontrolled progression of the complications. To stop the progression of these complications and to repair the damage, we would need to either replace the damaged ECs or create brand new vascular networks. Considering mounting evidence of stem/progenitor cells in various tissues including the blood vessel wall [40], we can speculate that the reason diabetic patients exhibit impaired repair mechanisms is because these stem/progenitor cells are also affected.

## Vascular stem cells (VSCs): current evidence and promise

Stem cells are defined by their ability to both self-renew and differentiate into functionally mature cells [41]. The potential of the cells is determined by the hierarchy and specialization level. Stem cells have been identified in a variety of post-embryonic tissues, including bone marrow, blood, fat, and skin [42,43]. Finding these stem cell populations presents the opportunity for non-invasive tissue repair and tissue regeneration including the vascular tissue. In terms of regenerating brand new vascular networks (*de novo* formation), we must first find a suitable cell source. Ideally, it would be one cell type or a subpopulation that can produce both endothelial cells and the supportive perivascular cells. The notion of a specific *asculartemell* (VSC) able to produce mature/functional cells of the blood vessels is slowly gaining momentum. Several groups have demonstrated the existence of a common vascular precursor cell in both mouse and human studies. Kattman et al. used cell tracing studies in mice and showed that cardiomyocytes arise from a cell population expressing the VEGF receptor-2 (VEGFR2/Flk1) [44], indicating that they develop from a progenitor that also has vascular potential. They followed up these studies with an embryonic stem cell differentiation model, in which they isolated cardiovascular progenitors (brachyury+; VEGFR2+) from human embryoid bodies (EBs) and successfully demonstrated the potential for generating cardiomyocytes, endothelial cells, and vascular smooth muscle cells [44]. Yamashita and colleagues showed that VEGFR2+ cells, derived from embryonic stem cells, could differentiate into both endothelial and mural cells through differing culture conditions [45]. These cells were also able to reproduce the vascular organization process when placed in three-dimensional culture systems [45]. Similarly, Ferreira et al. demonstrated that vascular progenitors (CD34+) isolated from EBs will give rise to endothelial and smooth muscle-like cells, and have the ability to form vascular networks when implanted *in vivo*[46].

The exact identity of the VSCs is still not clear. There is ample evidence that these VSCs are found in the bone marrow and circulation and are quite distinct from hematopoietic stem cells. Selection of CD133+ cells from the circulation purifies a population(s) of cells that under different culture conditions, will produce lineage-restricted endothelial progenitor cells (EPCs) and mesenchymal/mesodermal progenitor cells (MPCs) [47-51]. However, cells expressing pan hematopoietic marker CD45 fail to yield endothelial cells [52-54]. It is unknown thus far whether one or more stem cell subtypes reside within this CD133+ population that are limited in their capacity to produce endothelial and mesenchymal cell types. More importantly, stem cell-derived EPCs and MPCs form functional vascular networks [45,46,52]. Whether this is a feasible avenue for diabetic patients is just recently being probed.

## Endothelial progenitor cells (EPCs) and diabetic complications

**Figure 4:**
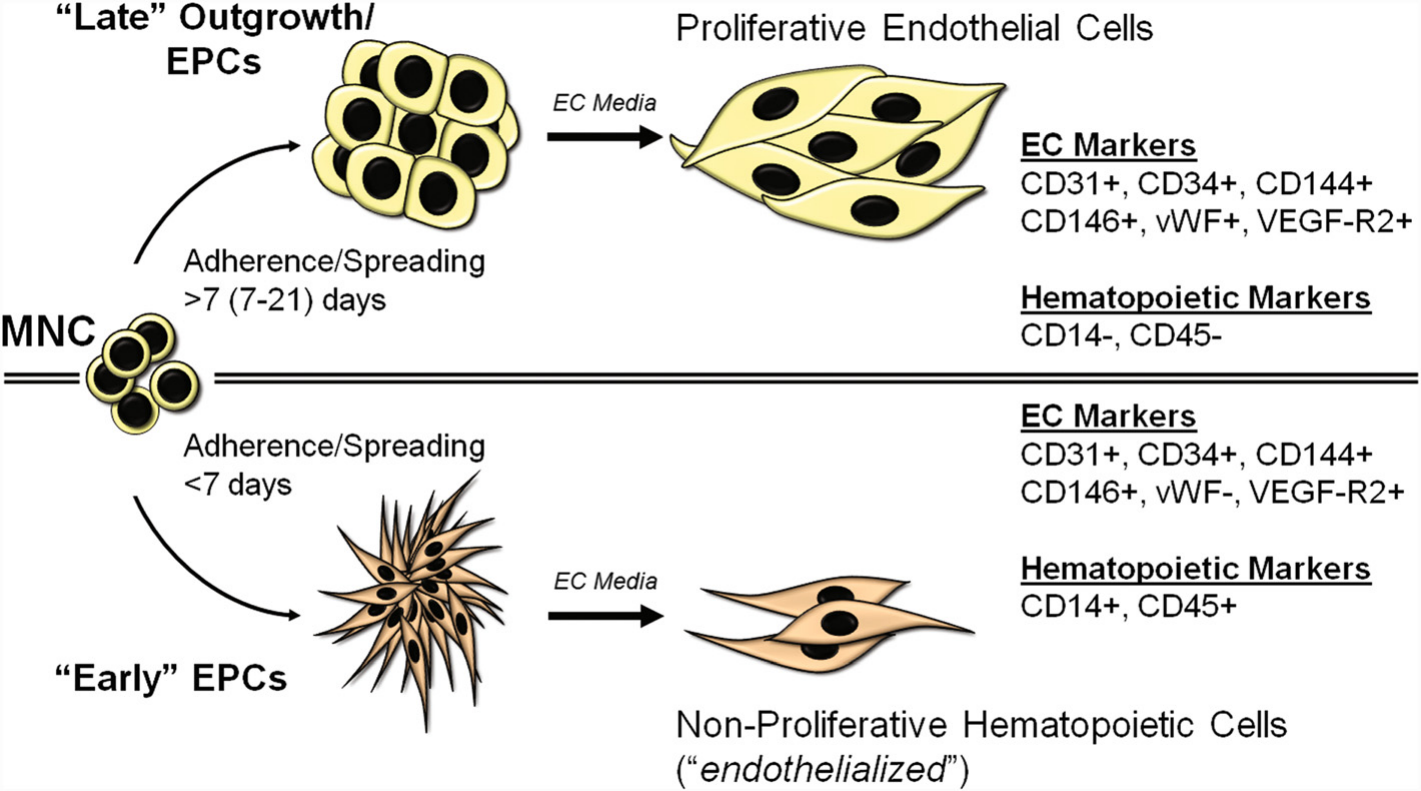
**Early and Late EPCs.** Schematic illustrating the two major types of cell colonies arising from blood and bone marrow mononuclear cells. Short term colonies appear within 7days of culture and are comprised of spindle shape or polymorphic cells. These early EPCs (also called angiogenic EPCs) express a number of endothelial and hematopoietic markers but fail to proliferate in culture. Late colonies, appearing from 721days, are comprised of epitheloid cells with high proliferative capacity. These late EPCs (also called vasculogenic EPCs) express all markers of mature endothelial cells but lack hematopoietic marker expression.

Progenitor, or precursor, cells are committed (lineage-restricted) and highly proliferative derivatives of stem cells. These cells may be capable of doubling their population every 1015 hours [55]. However, unlike fully mature cells, progenitors may express markers of full maturity in addition to select stem cell markers [55]. For example, EPCs share markers of both stem and mature endothelial lineages [56]. With the potential use of EPCs, either clinically or as a biomarker, accurate identification and reproducible classification is of great importance. Despite advances in research on this subject, a lack of consensus remains on how EPCs should be defined. Traditionally, EPCs have been identified as the spindle-shaped or polymorphic cells that appear within 24days in culture after isolation of the mononuclear cell (MNC) fraction from blood or bone marrow [57] (Figure 4). The cells are characterized by Ulex europaeus agglutinin binding and DiI-labeled acetylated-low density lipoprotein (LDL) uptake [58-60]. These two properties are considered functional characteristics of ECs. However, along with expressing some EC markers, these short-term EPC colonies also express monocyte-specific marker CD14 and/or hematopoietic cell marker CD45. Further, acetylated-LDL uptake is a known feature of monocytes which was identified in 1979 [61]. Ulex europaeus agglutinin is a lectin which binds to the EC surface via fucose resides. Also, these fucose residues are not specific to the ECs [62].

**Figure 5:**
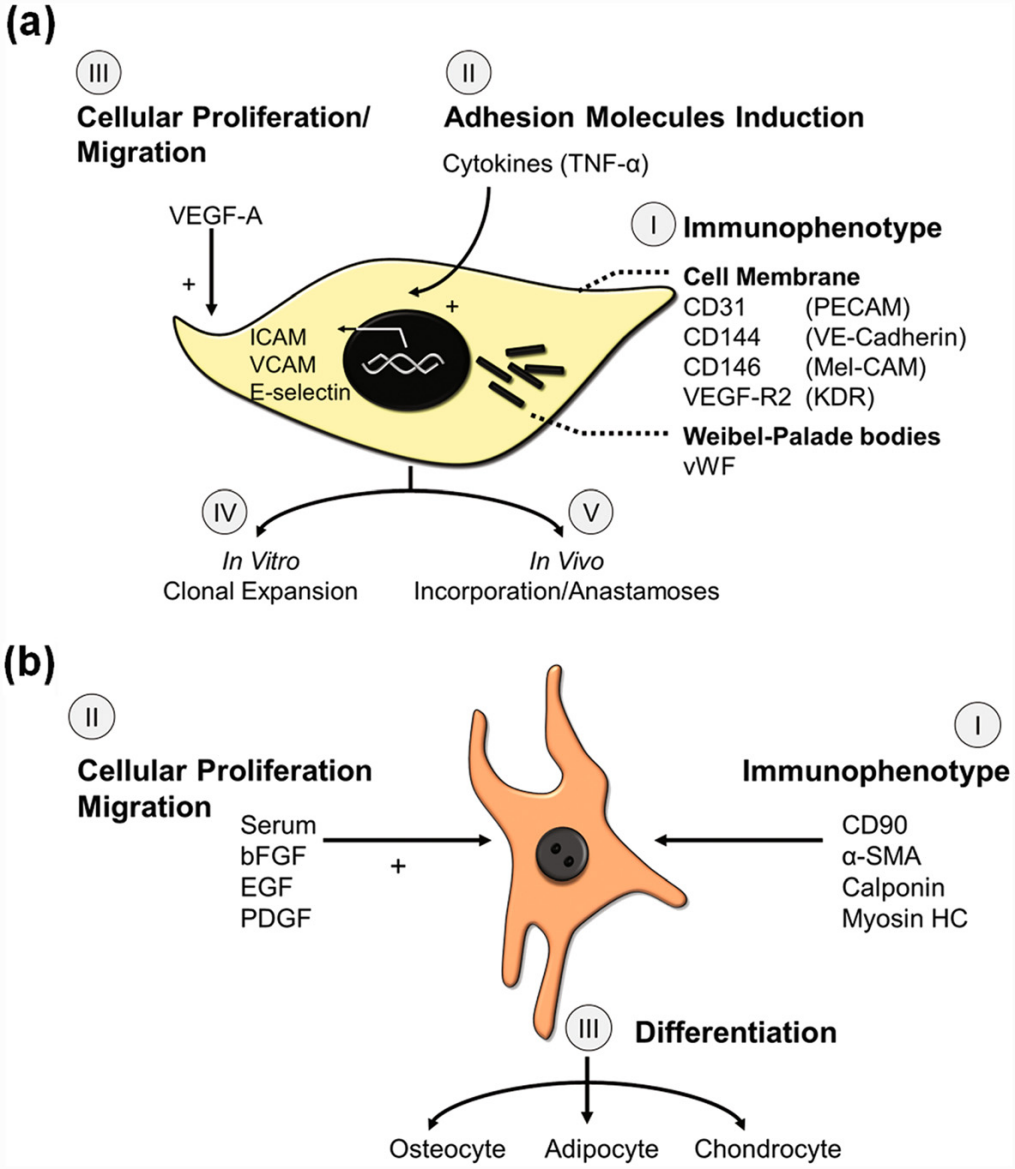
**Characterization scheme for EPCs and MPCs.(a)** EPCs are defined by a set of morphological and phenotypic characteristics. These cells show properties of bone fide endothelial cells including expression and localization of CD31 and ve-cadherin on the cell membrane and von willebrand factor in the Wieble Palade bodies. Vascular endothelial growth factor is a mitogen for endothelial cells and EPCs. Also, EPCs induce adhesion molecules when challenged with cytokines, similar to mature endothelial cells. The progenitor properties include cloncal growth potential and in vivo vasculogenesis. **(b)** MPCs are also defined by morphological and phenotypic characteristics of mature mesenchymal cells (such as perivascular cells) and mesodermal progenitors. Similar to mature smooth muscle cells, MPCs express CD90, -smooth muscle actin, and calponin. Upon treatment with specific growth factors, such as plateled-derived growth factor and epidermal growth factor, MPCs proliferate and also exhibit chemotaxis. The progenitor phenotype involves the ability of the cells to give rise to mesenchymal lineage-specific cells such as adipocytes, osteocytes, and chondrocytes.

So what are EPCs? The single most important property (or functional attribute) of EPCs is the ability to incorporate into blood vessels. In other words, EPCs are *vasculogenic*. In comparison, early EPCs may be considered *angiogenic* as they may facilitate angiogenesis through elaboration of growth factors. We and others have characterized marker expression and cellular activities of vasculogenic EPCs extensively. EPCs display properties of both ECs and unipotent progenitor cells. EPCs differ from mature ECs in CD133 expression (positive on EPCs but readily lost upon culture) [48,49], proliferation/growth kinetics (EPCs show lower population doubling time and higher cumulative population doublings) [53,63], and response to endostatin (EPCs are stimulated whereas mature ECs are inhibited) [48]. Over time, EPCs resemble mature ECs in terms of marker expression and all cellular activities [48,52,53]. Much of the controversy behind angiogenic and vasculogenic EPCs could be negated by performing functional cellular activity tests (summarized in Figure 5). These include assessing the expression of endothelial-specific markers [48,52,53], activation by cytokine challenge [48], and most importantly, the ability of the cells to create blood vessels [52,53].

EPCs may be involved in vascular dysfunction in chronic diabetic complications (reviewed in [64]). It has been demonstrated that type 1 and type 2 diabetics maintain a lower circulating number of EPCs when compared with healthy subjects [56,65-67]. In two similar studies, flow cytometric analysis was used to quantify EPCs (CD34 ^+^/VEGFR2^+^/CD31^+^) in diabetic patients. These studies showed that EPCs were reduced by 44% and 40%, respectively [65,68]. More recently, the number of circulating CD34^+^/VEGFR2^+^ cells were shown to correlate with glycemic control in type 2 diabetic patients [69]. This study also highlighted the negative relationship between circulating CD34^+^/VEGFR2^+^ cells and arterial stiffness in diabetic patients. Since these surface markers are not exclusive to EPCs, the reduced number may be inclusive of altered levels of hematopoietic stem/progenitor cells. In fact, a fairly large study with 120 patients with ischemic heart disease showed reduced levels of bone marrow-derived CD34^+^/CD45^+^ cells which also correlated with glycated hemoglobin HbA1c levels [70]. *In vitro* experimental studies using early EPCs have also shown a lower angiogenic ability [65], and impaired adherence to the mature EC monolayer in diabetes [71,72]. Though less work has been done on the late vasculogenic EPCs, we have shown that high levels of glucose do not alter the cell growth, proliferation, or migration of late EPCs [73,74]. The identical condition, however, increased ET receptor expression in the mature ECs and enhanced glucose-induced mature EC death. These findings show vasculogenic EPCs may be resistant to the adverse effects of high glucose.

Though EPC number may be reduced in long-term diabetes, there is still promise for their therapeutic potential. If the cellular activity of the EPCs remains intact in a diabetic setting, administration of *ex vivo* expanded EPCs should essentially work to improve vascular dysfunction. Not only has successful expansion of adult blood-derived EPCs been shown *in vitro*, but their ability to form fully functional vascular networks has also been demonstrated *in vivo*[47,53]. It is important to note that in order to form stable and durable networks, EPCs require co-implantation with a source of perivascular cell. MPCs, being derived from the same CD133+ fraction as EPCs, may be a suitable candidate for this task.

## Mesenchymal progenitor cells (MPCs) and diabetic complications

MPCs are multipotent cells that are derived, along with EPCs, from the CD133+ population of circulating cells [51]. MPCs can be isolated in large quantities from adult human bone marrow [75]. In addition, MPCs have been identified in liver [76], spleen [76], and adipose tissue [77]. Like EPCs and other progenitor cell types, MPCs share properties of both stem cells and mature cells. MPCs can be characterized by a combination of phenotypic and functional properties, including expression of cell surface markers, cell adhesion molecules, and differentiation potential (Figure 5). Because there is not one marker that is specific to MPCs, all parameters must be taken into consideration when properly identifying this cell population. Mesenchymal cells in culture typically exude a spindle-like morphology [75], however, some heterogeneity has been noted depending on the tissue source and especially when arising from differing species. MPCs are negative for both the endothelial marker CD31 and the hematopoietic marker CD45 [52]. Analysis of mRNA and/or protein can be used to demonstrate expression of -smooth muscle actin (SMA), calponin, CD90, PDGFR, and NG_2_[47,52]. Functionally, MPCs differentiate into the mesenchymal lineage cells including adipocytes, osteocytes, and chondrocytes [47,52,75].

Not much is known about a possible pathogenic role of MPCs in diabetic complications. In terms of therapeutic benefit, however, recent studies show improvement and amelioration of complications, including cardiomyopathy, nephropathy, neuropathy, and wound healing by MPCs. Using a rat model of diabetic cardiomyopathy, MPCs were administered intravenously and shown to attenuate cardiac remodeling and improve myocardial function through a marked increase in the activity of matrix metalloproteinase (MMP)-2 and decrease in MMP-9[78]. In addition, reduced levels of VEGF, insulin-like growth factor-1 (IGF-1), adrenomedullin (AM), and hepatocyte growth factor (HGF) were found [78]. The MPCs differentiated into both cardiomyocytes and vascular ECs, improving myocardial perfusion and regeneration in the diabetic heart [78]. MPCs have also successfully improved diabetic nephropathy in mice. After systemic injection, the precursor cells were shown to engraft in the damaged kidneys and differentiate into renal cells, improving renal function and the regeneration of glomerular structures [79,80]. Furthermore, MPCs improve diabetic polyneuropathy through increased secretion of angiogenic cytokines such as bFGF and VEGF when injected intramuscularly [81]. In a model of skin wound healing, administration of MPCs in streptozotocin-induced diabetic rats completely normalized the delayed wound closure time [82]. This effect was mediated, in part, through reduced number of infiltrating CD45^+^ cells into the wounds. This study involved normal MPCs (i.e. cells isolated from non-diabetic rats) and the question remains whether diabetes causes alteration of the functional properties of MPCs. This is a new field of research with not much known. However, a recent study showed that AGEs (Figure 2) may increase the generation of reactive oxygen species and reduce the proliferation and migration of MPCs [83]. Whether this plays a role in human diabetes or in animal models of diabetic complications, requires further studies.

Given the advantages that MPCs have over other cell types (differentiation potential and capability for regulation of the immune response), they are likely to be good therapeutic candidates in diabetic complications. The recent studies in animal models do show promise. Several studies have also reported that treatment with MPCs can enhance angiogenesis through paracrine effects [84-86]. The paracrine role may involve the release of angiogenic factors to facilitate EPC homing and the restructuring of vascular networks [52].

## Concluding remarks

**Figure 6:**
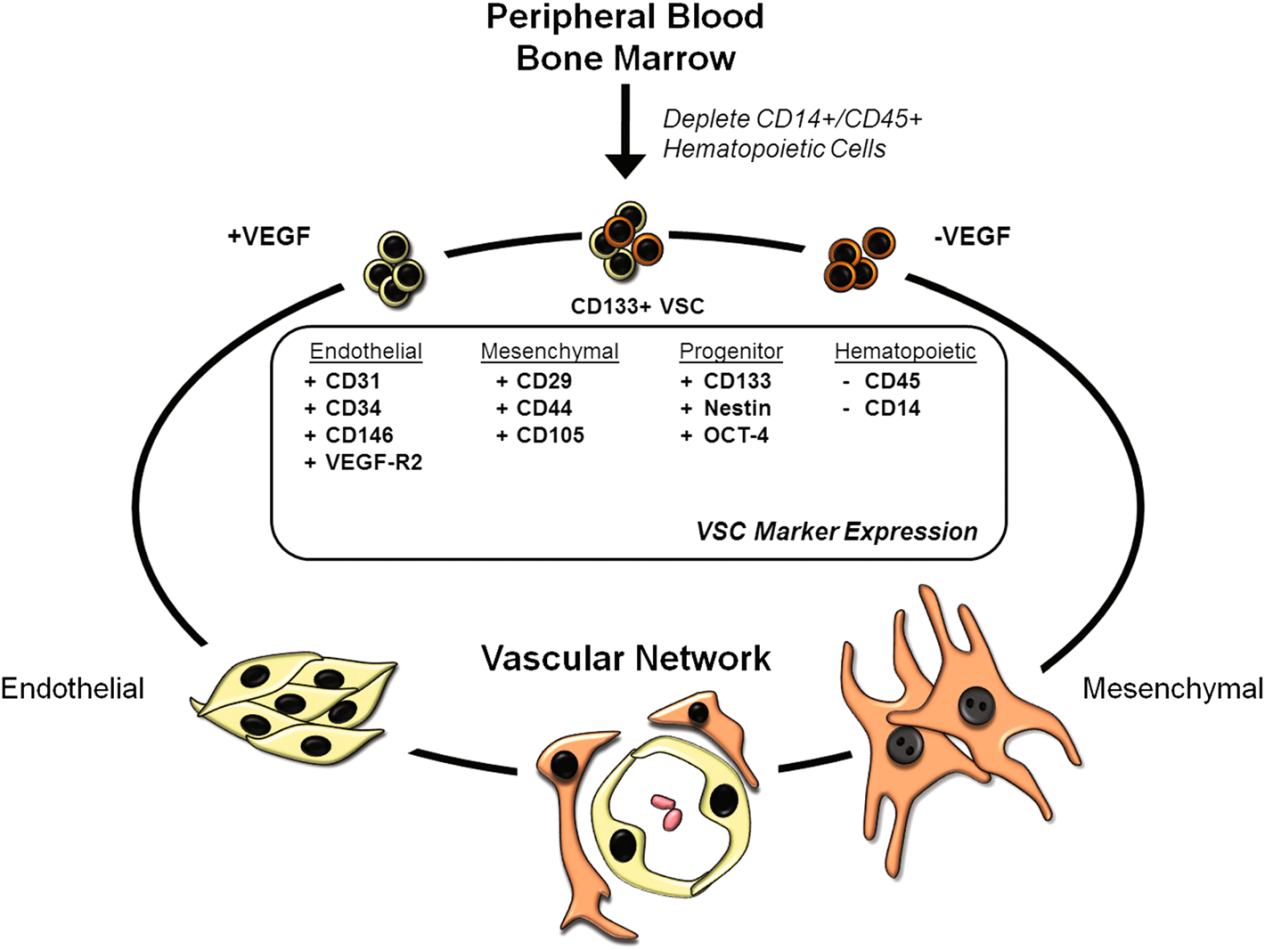
**The potential of VSCs for therapeutic use in diabetics.** A schematic of our working hypothesis showing that CD133+ VSCs are non-invasively isolated from diabetic patients and differentiated into endothelial and mesenchymal lineages by defined media. EPCs and MPCs are then expanded *ex vivo* and re-implanted in the patients to repair the damage and restore vascular homeostasis [middle box shows the immunophenotype of VSCs; VEGF=vascular endothelial growth factor (obligatory factor for endothelial lineage)].

Examining the long-term effects of diabetes has led to implication of vascular ECs as the primary target of hyperglycemia-induced damage. Subsequently, entire vascular networks in target organs become dysfunctional and provide the foundation for the complications we see in the patients. Experimental evidence shows that stem/progenitor cells isolated from diabetic mice are able to restore vascular homeostasis [87,88]. This suggests that the main stem cell deficit in diabetes is reduced number. This reduction may take place somewhere between the bone marrow and circulation. If we can find a way utilize these two cells types to repair vascular damage and restore blood vessel functioning, there is hope that the chronic complications can be attenuated (Figure 6).

The success of therapeutic vascularization will rely on many factors, one of which being the ability of engineered blood vessels to form stable and functional anastomoses with the host vasculature. Neovascularization has been successfully shown thus far using human umbilical vein endothelial cells (HUVECs), as well as human microvascular endothelial cells (HDMECs) [89,90]. However, there are limitations to the clinical use of these particular EC types because of the lower yield. Considering the ease with which EPCs can be isolated from adult peripheral blood, an opportunity is presented to obtain these cells non-invasively and in large enough quantities for expansion *ex vivo*. Following expansion, the cells can be implanted into the diabetic patient to restore vascular homeostasis. It has previously been shown that adult and cord blood-derived EPCs have the ability to form functional vascular networks *in vivo*[53,91]. Importantly, this requires co-implantation of perivascular cells in order to maintain stable, functional networks. MPCs are an ideal candidate for a viable source of perivascular cell because, like EPCs, they can be isolated with minimal complications from sites such as bone marrow [75] and even adult blood [92]. We have previously shown the success of subcutaneous co-implantation of EPCs and MPCs into the backs of athymic *nu/nu* mice, resulting in the creation of human microvessels that formed functional anastomoses with the host vasculature [52].

## Abbreviations

AGE = Advanced glycation end products; AM = Adrenomedullin; DCCT = Diabetes Control and Complications Trial; EB = Embryoid body; EC = Endothelial cell; ECM = Extracellular matrix; EPC = Endothelial progenitor cell; ET = Endothelin; Glut = Glucose transporter; HDMEC = Human dermal microvascular endothelial cell; HGF = Hepatocyte growth factor; HUVEC = Human umbilical vein endothelial cell; IGF = Insulin-like growth factor; LDL = Low-density lipoprotein; MAPK = Mitogen-activated protein kinase; MMP = Matrix metalloproteinase; MPC = Mesenchymal progenitor cell; NO = Nitric oxide; PARP = Poly (ADP-ribose) polymerase; PDGFR = Platelet-derived growth factor receptor; ROS = Reactive oxygen species; UKPDS = United Kingdom Perspective Diabetes Study; VEGF = Vascular endothelial growth factor; VEGFR = VEGF receptor; VSC = Vascular stem cell; vWF = von Willeband Factor.

## Competing interests

The authors confirm that there are no competing financial interests.
